# A comparison of mRNA sequencing (RNA-Seq) library preparation methods for transcriptome analysis

**DOI:** 10.1186/s12864-022-08543-3

**Published:** 2022-04-13

**Authors:** Hiroki Ura, Sumihito Togi, Yo Niida

**Affiliations:** 1 Center for Clinical Genomics, Kanazawa Medical University Hospital, 1-1 Daigaku, Uchinada, Kahoku, Ishikawa 920-0923 Japan; 2 Division of Genomic Medicine, Department of Advanced Medicine, Medical Research Institute, Kanazawa Medical University, 1-1 Daigaku, Uchinada, Kahoku, Ishikawa 920-0923 Japan

**Keywords:** Transcriptome, RNA-Seq, Gene expression, Alternative splicing

## Abstract

**Background:**

mRNA sequencing is a powerful technique, which is used to investigate the transcriptome status of a gene of interest, such as its transcription level and splicing variants. Presently, several RNA sequencing (RNA-Seq) methods have been developed; however, the relative advantage of each method has remained unknown. Here we used three commercially available RNA-Seq library preparation kits; the traditional method (TruSeq), in addition to full-length double-stranded cDNA methods (SMARTer and TeloPrime) to investigate the advantages and disadvantages of these three approaches in transcriptome analysis.

**Results:**

We observed that the number of expressed genes detected from the TeloPrime sequencing method was fewer than that obtained using the TruSeq and SMARTer. We also observed that the expression patterns between TruSeq and SMARTer correlated strongly. Alternatively, SMARTer and TeloPrime methods underestimated the expression of relatively long transcripts. Moreover, genes having low expression levels were undetected stochastically regardless of any three methods used. Furthermore, although TeloPrime detected a significantly higher proportion at the transcription start site (TSS), its coverage of the gene body was not uniform. SMARTer is proposed to be yielded for nonspecific genomic DNA amplification. In contrast, the detected splicing event number was highest in the TruSeq. The percent spliced in index (PSI) of the three methods was highly correlated.

**Conclusions:**

TruSeq detected transcripts and splicing events better than the other methods and measured expression levels of genes, in addition to splicing events accurately. However, although detected transcripts and splicing events in TeloPrime were fewer, the coverage at TSS was highest. Additionally, SMARTer was better than TeloPrime with regards to the detected number of transcripts and splicing events among the understudied full-length double-stranded cDNA methods. In conclusion, for short-read sequencing, TruSeq has relative advantages for use in transcriptome analysis.

## Background

The large majority of human genes are transcribed as pre-mRNAs that include exons and introns, which then produce a mature mRNA following removal of introns during splicing events [1]. Various mRNA products can thus be generated through posttranscriptional alternate intron splicing to produce structurally and functionally different protein isoforms [2]. Approximately 90% of human genes undergo alternative splicing, of which 40% of the human protein-coding genes generate multiple protein isoforms [3–5]. Several alternative splicing events, including alternative 5′ or 3′ splicing site usage, exon skipping, intron retention, and mutually exclusive exons have been reported [6–8]. These alternative splicing events therefore produce different mRNAs that translate to different protein isoforms with distinct coding sequences. In turn, during development and cell differentiation, these alternative splicing events control various expression patterns of multiple genes [9–13]. However, it is reported that several splicing events can be associated with specific pathology or are pathogenic themselves [14–16]. Thus, although researchers need to investigated alternative splicing repertoire at the transcription level, the RNA sequencing (RNA-Seq) method has not been well determined.

**Fig. 1:**
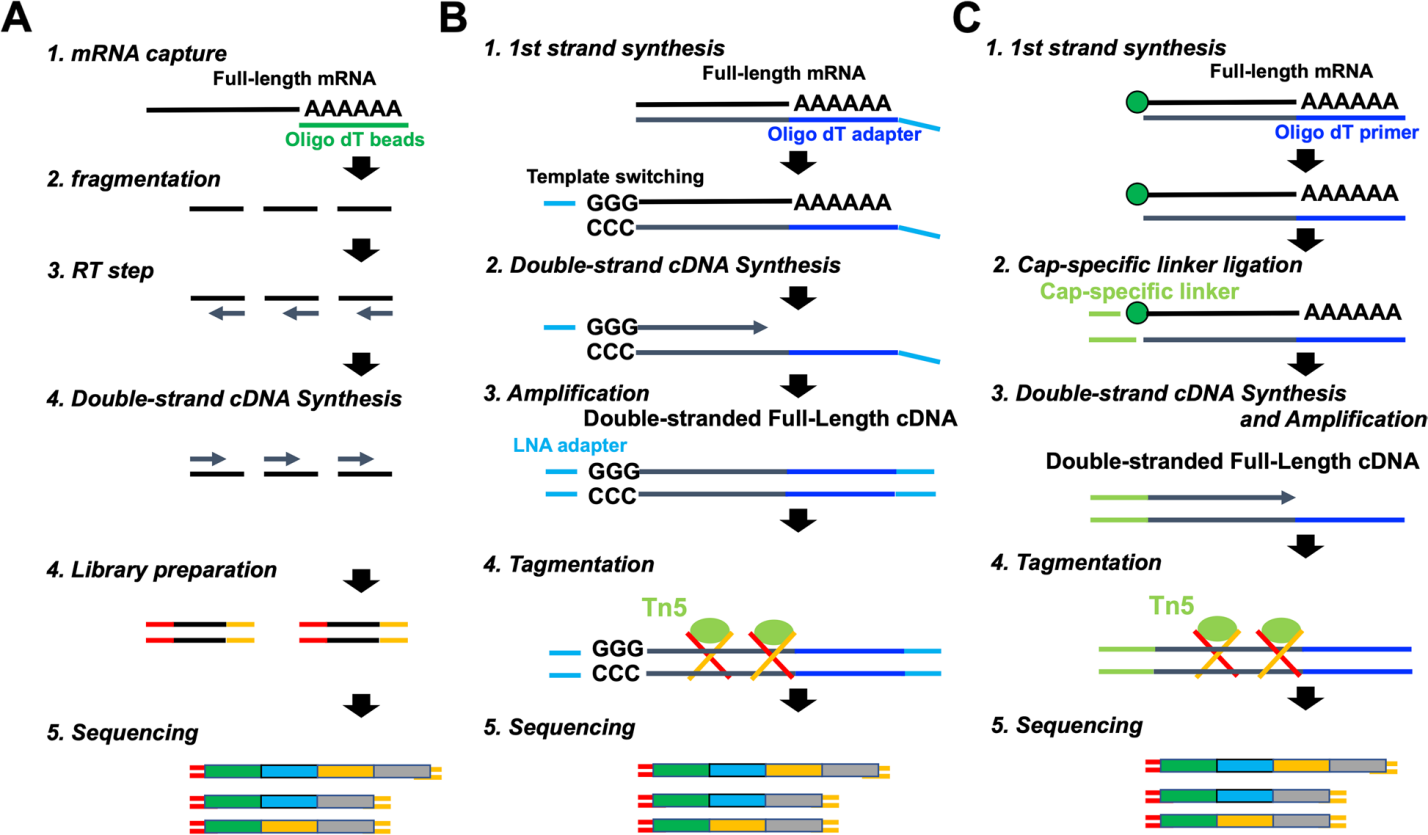
Library preparation steps. **A** Workflow for the TruSeq method (**B**) Workflow for the SMARTer method (**C**) Workflow for the TeloPrime method

RNA-Seq is a powerful tool to quantify and characterize the transcriptome [17]. So far, RNA-Seq is primarily used to quantify the expression level and relative changes in gene expression patterns between samples [18]. It also detects novel and previously known splice variants accurately. Presently, several RNA-Seq methods have been developed and established, resulting in several choices for researchers. During the traditional RNA-Seq method (TruSeq), captured mRNAs using oligo dT beads are sheared randomly into fragments, then reverse transcribed into cDNAs. Afterward, double-stranded cDNAs are generated from these cDNA transcripts (Fig. 1). In contrast, the full-length double-stranded cDNAs are generated without fragmentation during SMARTer and TeloPrime methods [19, 20]. As observed, the SMARTer method takes advantage of template switching using the MMLV (Moloney Murine Leukemia Virus) reverse transcriptase enzyme to generate full-length double-stranded cDNAs. However, the TeloPrime method takes advantage of the cap-specific linker ligation to generate a complete full-length double-stranded cDNAs from complete 5′ capped mRNA molecules.

On the basis of the reports above, several studies have investigated the performance of different RNA-Seq methods for quantification of transcriptome [21, 22]. However, there were not many investigations on the proficiency of these methods for use in alternative splicing analysis. Here, we compared the performance of three commercially available RNA-Seq library preparation kits, the traditional RNA-Seq method (TruSeq), in addition to full-length double-stranded cDNA methods (SMARTer and TeloPrime) to determine the best method for transcriptome analysis, including quantification and alternative splicing analyses.

## Results

### Comparison between TruSeq, SMARTer, and TeloPrime for quantification analysis

**Fig. 2:**
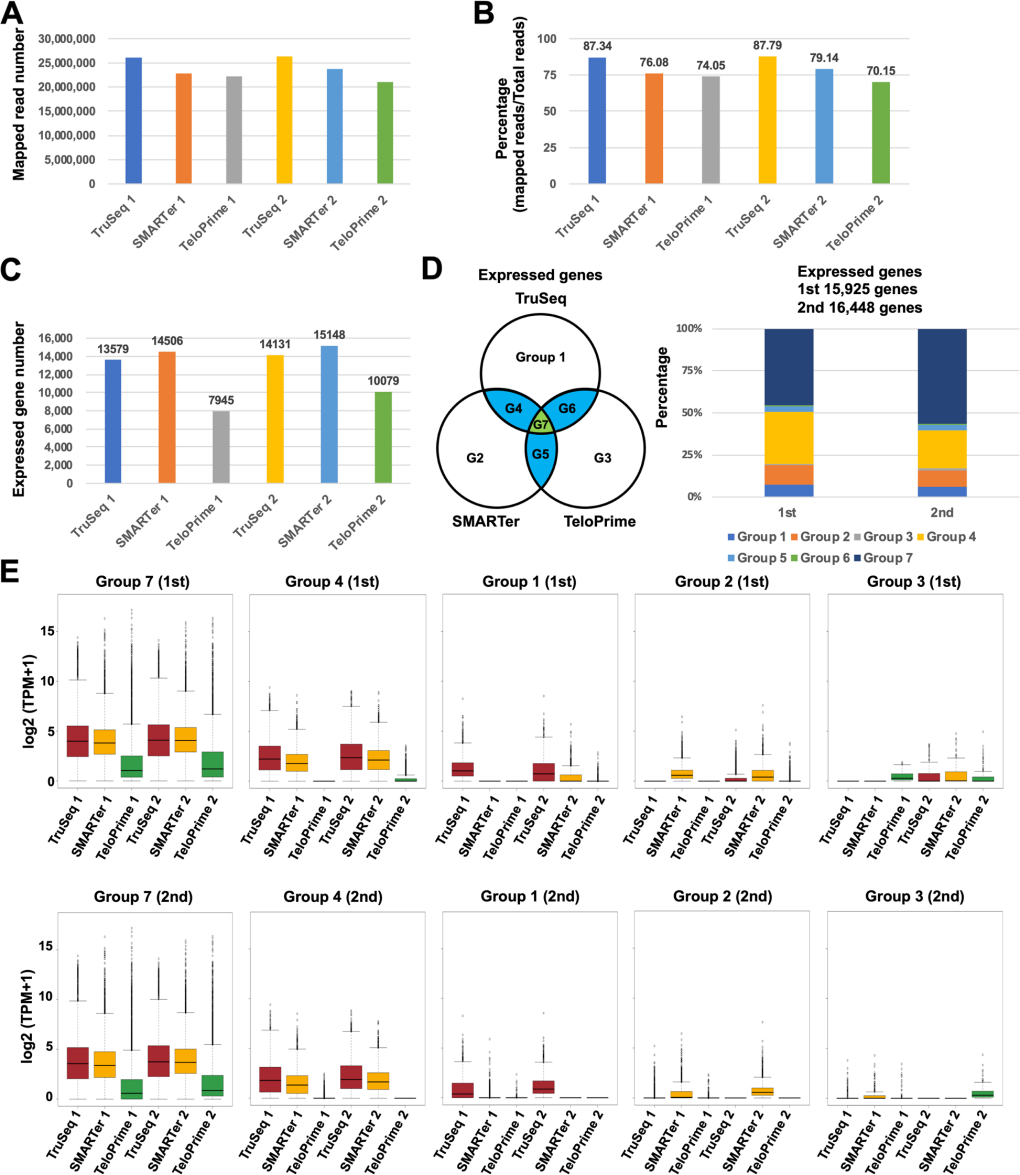
Comparison between TruSeq, SMARTer, and TeloPrime methods for quantification analysis. **A** The mapping read number. **B** Percentage of mapped reads. **C** Number of detected expressed genes (TPM (Transcripts per million) > 0). **D** The percentage of expressed genes in each group. **E** Boxplot of expressed genes in each group

**Fig. 3:**
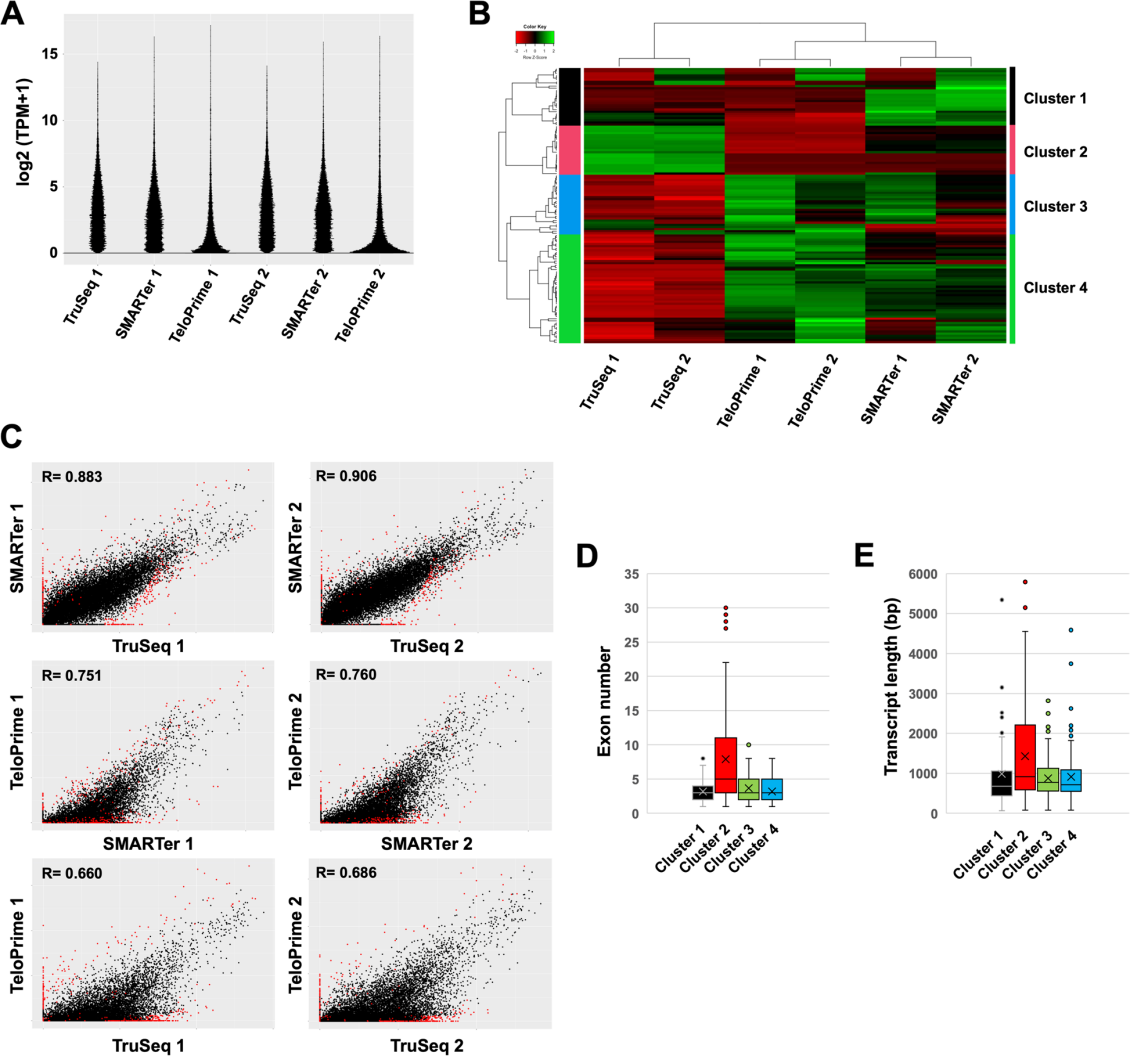
Comparison of each expressed gene between TruSeq, SMARTer, and TeloPrime methods. **A** Violin plot of expressed genes. **B** Heat map of hierarchical clustering of 129 differential expressed genes (FDR < 0.05) between two samples using TruSeq, SMARTer, and TeloPrime. **C** A scatterplot [log2 (TPM + 1)] of total genes (26,475 genes). Red spots indicate the differential expression of genes (*p* < 0.05). **D** Boxplot of exon number in each cluster (129 differential expressed genes). **E** Boxplot of transcription length in each cluster (129 differential expressed genes)

To evaluate the performance of adopted RNA-Seq methods in quantification analyses, we compared their accuracies of gene detection, including expression patterns of TruSeq, SMARTer, and TeloPrime using these two samples (of the peripheral blood mononuclear cells) from two patients. The number of paired mapping reads and percentages of mapped reads was similar between the three methods (Fig. 2A and B). The number of detected expressed genes was also similar in TruSeq and SMARTer (Fig. 2C). The number of genes detected in TeloPrime was less than approximately half that of the TruSeq and SMARTer. Moreover, the hierarchical clustering analysis showed that the expression pattern was highly correlated with same methods despite different patient’s samples, indicating that the difference in methods was more significant than differences between the samples. (Fig. 3B). The expression pattern between TruSeq and SMARTer was also strongly correlated (*R* = 0.883 and 0.906) (Fig. 3C). Alternatively, the expression pattern of TeloPrime recorded a relatively low correlation (*R* = 0.660 to 0.760) because the expression level in TeloPrime was lower than that of the TruSeq and SMARTer. The results also showed that expression levels of cluster 2 genes in TruSeq was higher than that of SMARTer and TeloPrime (Fig. 3B). Besides, cluster 2 genes had more exons and longer transcripts than other clusters (Figs. 3D and E). On the other hands, the expression levels of cluster 3 and 4 genes in SMARTer and TeloPrime was higher than that of TruSeq. Moreover, the expression level of cluster 1 genes in TeloPrime was higher than TruSeq and SMARTer. The cluster 1, 3 and 4 genes had relatively shorter transcripts than the average length of human transcripts, indicated that RNA-Seq methods (SMARTer and TeloPrime), which generated full-length double-stranded cDNAs, had a disadvantage during cDNA synthesis of long transcripts.

While the expression level was similar in TruSeq and SMARTer, the expression level in TeloPrime was lower than that in the TruSeq and SMARTer (Fig. 3A and C). Furthermore, we compared the detected genes between the three different RNA-Seq methods (Fig. 2D). Many detected genes were in Groups 7 and 4, indicating that detected genes were commonly detected especially between TruSeq and SMARTer, whereas about one-third of common genes were undetected in TeloPrime. The Gene Ontology (GO) enrichment analysis showed that the gene sets of Group 4 and 7 are the peripheral blood mononuclear cells associated genes. Next, we investigated whether method-specific detected genes were detected in each method (Fig. 2E). As observed, the expression level of genes detected in method specific groups (Groups 1, 2, and 3) was lower than those of overlapping groups (Groups 7 and 4). We observed, although several genes were undetected in both the first and second samples, these genes were detected in either sample. It seemed that some genes were stochastically but not method-dependently undetected due to low expression levels. Accordingly, these results suggested that the traditional RNA-Seq method (TruSeq) was better than SMARTer and TeloPrime for quantification analysis.

### Comparison between TruSeq, SMARTer, and TeloPrime for alternative splicing analysis

**Fig. 4:**
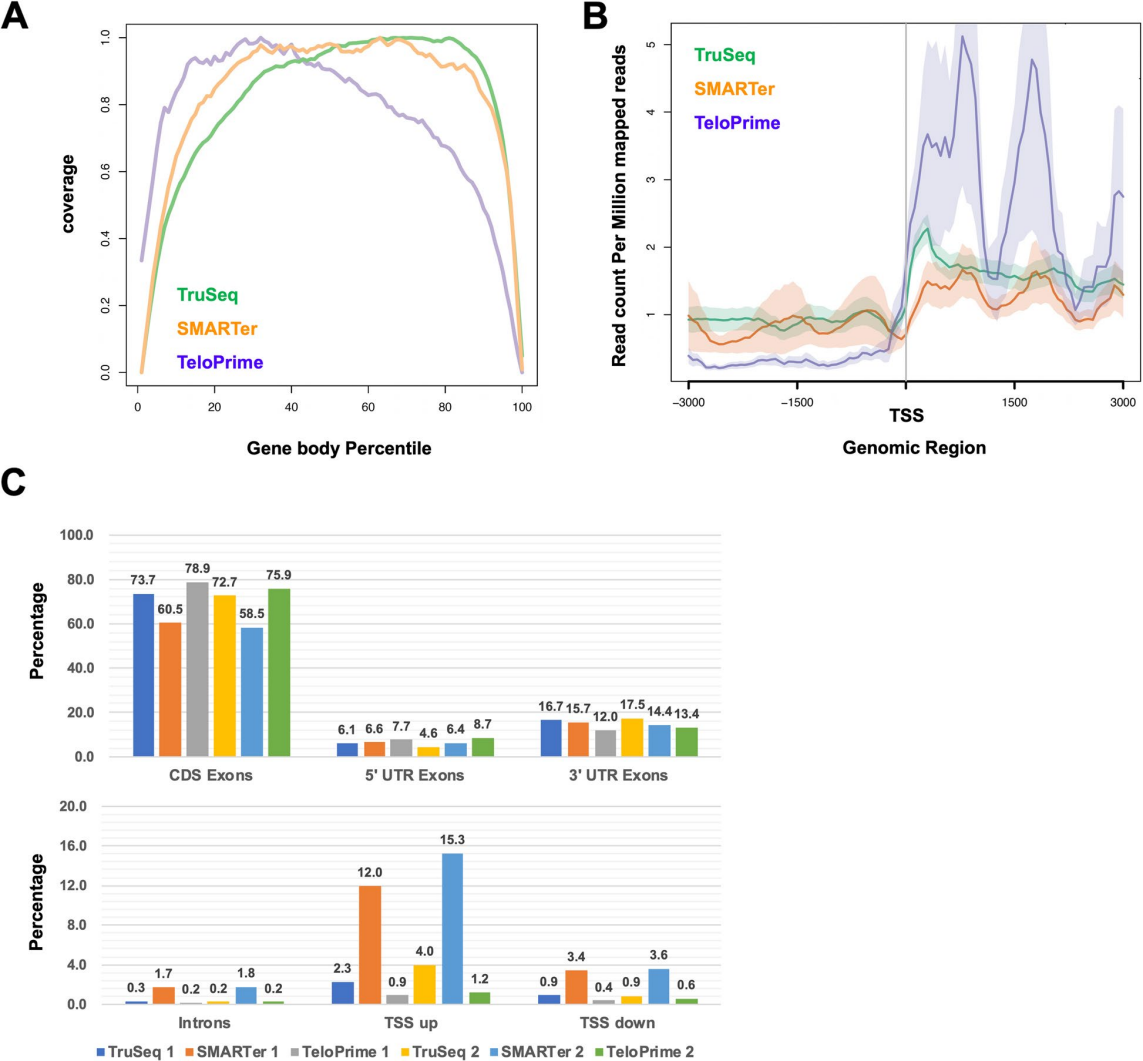
The distribution of mapped reads between TruSeq, SMARTer, and TeloPrime methods. **A** Distribution of the gene body was generated by RSeQC. **B** Distribution of TSS (Transcription start site) was generated by Ngsplot. **C** The percentage in each region [CDS Exons, 5′UTR Exons, 3′UTR Exons, Introns, TSS upstream (TSS up), and TSS downstream (TSS down)] were calculated by RSeQC

To evaluate the performance of RNA-Seq methods in alternative splicing analysis, we compared the coverage of transcript, enrichment of the transcription start site (TSS), and distribution of genomic regions. The coverage of transcripts from within the gene body showed that SMARTer was more uniform than others (Fig. 4A). Also, although the 5′ end coverage of TeloPrime was better, its 3′ end coverage was worse than others. From the results, the enrichment of TSS was higher in TeloPrime than in TruSeq and SMARTer (Fig. 4B). The distribution of coding exons (CDS exons) regions in SMARTer was slightly lower than that in the TruSeq and TeloPrime (Fig. 4C). Alternatively, the distribution of regions outside the gene’s body, such as introns, TSS upstream sites (TSS up), and TSS downstream sites (TSS down) in SMARTer was higher than TruSeq and TeloPrime, indicating that these mapped reads were amplified from genomic DNA in the SMARTer method. The 5′ untranslated region (UTR) of TeloPrime was also slightly higher than the TruSeq and SMARTer. Therefore, although these results propose that SMARTer uniformly covered the gene’s body, it also produced nonspecific genomic DNA amplification results. TeloPrime had the advantage of being able to investigate the TSS of transcripts, nonetheless, it had a disadvantage of inaccurately detecting other regions, except TSS.

**Fig. 5:**
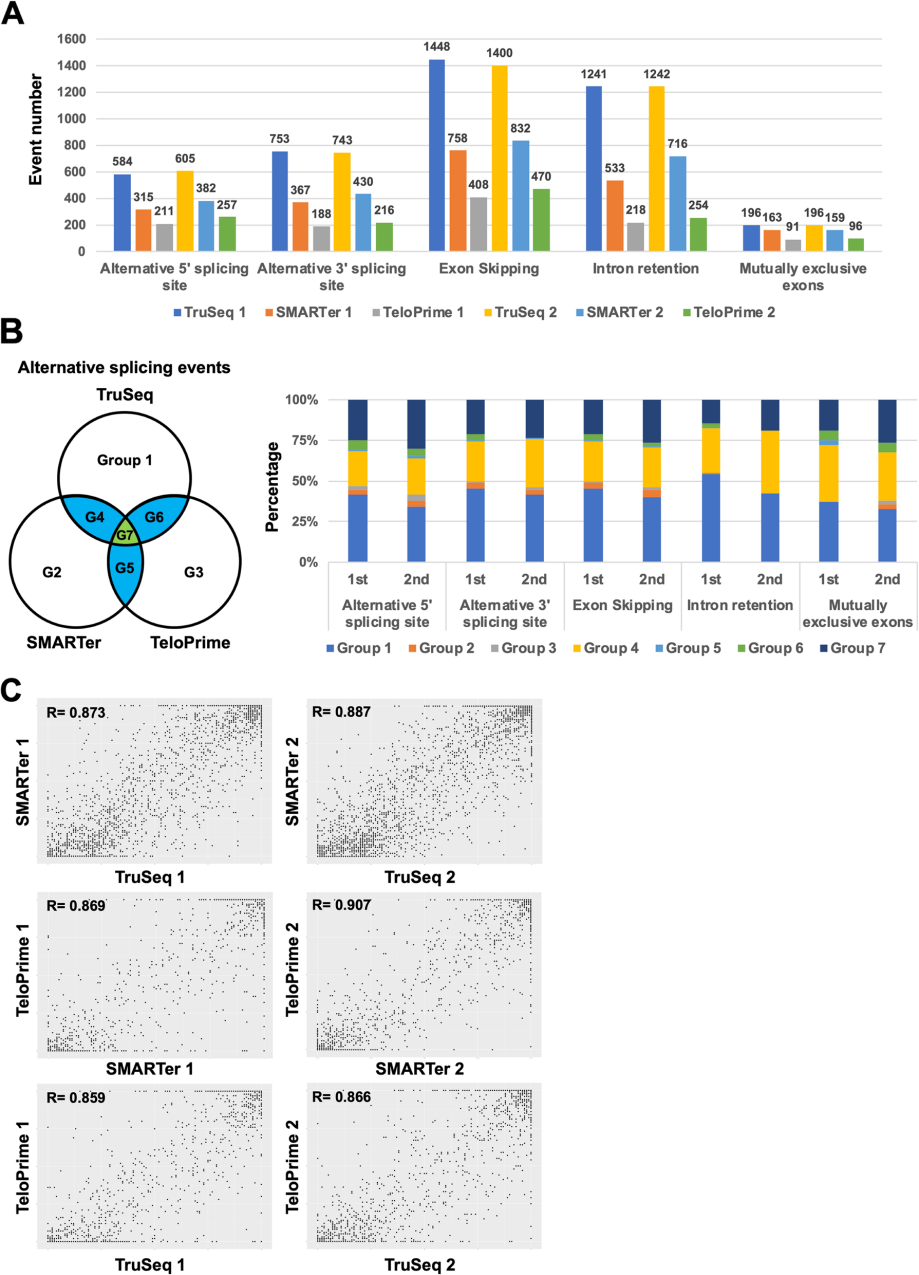
Comparison between TruSeq, SMARTer, and TeloPrime methods for the alternative splicing analysis. **A** The splicing event number per event (Alternative 5′ splicing site, Alternative 3′ splicing site, exon skipping, intron retention, and mutually exclusive exons). **B** The percentage of splicing events in each group. **C** A scatter plot showing the percent splicing index (PSI)

Next, we detected the alternative splicing events using SplAdder software and then compared the detection number and the quality of splicing event in TruSeq, SMARTer, and TeloPrime [23]. TruSeq detected about twofold more than detected by SMARTer and more than threefold detected by TeloPrime for alternative 5′ splicing site, alternative 3′ splicing site, exon skipping, and intron retention (Fig. 5A). With mutually exclusive exons, TruSeq detected about 1.3-fold more than SMARTer and twofold more than TeloPrime. Moreover, during any splicing events, half of the detected events were in Groups 7 and 4, indicating that detected events were commonly detected in TruSeq, SMARTer, and TeloPrime (Fig. 5B). The other half of the detected events were in Group 1, which was due to these splicing events detected using the TruSeq. Results also showed that the percent spliced in index (PSI) of the detected splicing events in common was that were highly correlated with TruSeq, SMARTer, and TeloPrime (Fig. 5C). PSI cannot be compared for method specific splicing events, but these results propose that the accuracy of PSI was the same for the three methods, at least for splicing events that can be detected in common. However, the traditional RNA-Seq method (TruSeq) was better than SMARTer and TeloPrime in terms of detected splicing event numbers obtained in alternative splicing analysis.

## Discussion

RNA-Seq based transcriptome analysis is a powerful tool for the quantification and detection of alternative splicing events and de novo splicing transcripts obtained from various genes during human disease and developmental studies. With the development and advancement of RNA-Seq methods, many library preparation methods and sequencing platforms have become available. However, most studies only have focused on the quantification of known transcripts. Here, we compared the performance of three commercially available RNA-Seq library preparation kits; the TruSeq, SMARTer, and TeloPrime to detect, which would be best for alternative splicing as well as quantification analyses.

During the quantification analysis, detected expressed genes were similar in TruSeq and SMARTer, however, detected genes in TeloPrime were about half. Furthermore, expression levels in TeloPrime were lower than that in the TruSeq and SMARTer. One reason for the decrease in the genes detected with TeloPrime is proposed to be that the efficiency of the cap-specific linker ligation was not sufficiently high, thereby making it is difficult to ligase the cap of many transcripts [24]. Although the expression pattern between TruSeq and SMARTer was highly correlated, genes in cluster 2 had a lower expression with SMARTer and TeloPrime than TruSeq. This finding is proposed to be because genes in cluster 2 had more exons and longer transcripts. Therefore, it was possible that the full-length double-stranded cDNA method, such as SMARTer and TeloPrime, had a bias, which resulted in the amplification difficulty observed with long transcripts compared with short transcripts. However, in the TruSeq, mRNA was first fragmented, then converted to the double-stranded cDNA by random primers to create a library. Thus, we propose that a uniform detection of gene expression and splicing events can be obtained regardless of the transcript’s length. Additionally, we observed that several genes were only detected using each method. Since the expression level of specific genes was lower than that of commonly detected, and specific genes detected in different samples of same methods, undetected genes were not method-dependently undetected but stochastically due to low expression. Considering these results, the traditional RNA-Seq method (TruSeq) is better than SMARTer and TeloPrime for quantification analysis.

During the alternative splicing analysis, TeloPrime detected TSS of transcripts better than TruSeq and SMARTer, however, the coverage of the region, except at the 5′ end in TeloPrime, was lower than that obtained using the TruSeq and SMARTer. Therefore, TeloPrime is proposed to be suitable for TSS analysis. Although detected splicing events in SMARTer were moderately lower than TruSeq, it is possible that nonspecific genome amplification affects alternative splicing analysis. Results showed that the detected splicing events number in TruSeq were more than in SMARTer and TeloPrime, whereas PSI of detected events were highly correlated with other methods. Thus, on the basis of the results, the traditional RNA-Seq method (TruSeq) is also proposed to be better than SMARTer and TeloPrime methods for alternative splicing analysis.

In this study, we compared the performance of three different methods using a short-read sequencer. Until now, RNA-Seq obtained with the short-read sequencer has been selected for transcriptome analysis due to its high fidelity, high coverage, and single-nucleotide resolution [25]. However, it is difficult to accurately characterize the full-length transcripts using short-read sequencers due to limitations of read length. Recently, long-read RNA sequencing methods, such as the PacBio and Oxford Nanopore Technologies sequencers have gained popularity due to its ability to overcome limitations of read lengths [26–32]. Consequently, although the TruSeq is better than SMARTer and TeloPrime during short-read sequencer, TruSeq is unavailable for the full-length transcriptome analysis using long-read sequencers due to its fragmented library. The full-length double-stranded cDNA methods, such as SMARTer and TeloPrime are therefore proposed to be required for transcriptome analysis using long-read sequencer.

## Conclusions

In this paper, we compared three commercially available RNA-Seq methods using the traditional method (TruSeq), in addition to full-length double-stranded cDNA methods (SMARTer and TeloPrime). We observed that TruSeq detected transcripts and splicing events better, and measured expression levels and splicing events more accurately. Although the performance of SMARTer was approximately similar to that of TruSeq, nonspecific genome DNA amplification occurred. Furthermore, since detected transcripts and splicing events in TeloPrime were fewer, the coverage at TSS was highest, indicating its suitability for TSS analysis. During short-read sequencing, we observed that the traditional method (TruSeq) had relative advantages for preferred use in transcriptome analysis. However, SMARTer is more useful for long-read RNA-Seq applications to determine the entire structure of mRNA transcripts than TeloPrime within the full-length double-stranded cDNA methods understudied.

## Methods

### Total RNA extraction

Total RNA from peripheral blood mononuclear cells, which were obtained from a patient with tuberous sclerosis complex, was extracted with TRIzol reagent (Thermo Fisher Scientific) according to the manufacturer’s instructions, as described previously [33]. Subsequently, the concentration and purity of isolated RNA molecules were measured spectrophotometrically (Nanodrop), after which the RNA integrity number was measured using TapeStation 4200 with a High Sensitivity RNA Screen Tape (Agilent Technologies, Santa Clara, CA).

### RNA-Seq library construction and library sequencing

The 100 ng of total RNA were used for RNA-Seq library construction. The fragmented double-strand cDNA was synthesized using TruSeq Stranded mRNA Library Prep Kit (Illumina, San Diego, CA, USA) according to manufacturer’s instructions. The library was amplified over 15 cycles and was constructed using KAPA Hyper Prep Kit (Kapa Biosystems, MA, USA) instead of TruSeq Stranded mRNA Library Prep Kit because we have the experience that KAPA Hype Prep Kit was better than TruSeq kit in the amplification efficiency. The full-length double-stranded cDNA was synthesized from total RNA using SMART-Seq v4 ultra-low input RNA kit (Takara Bio USA, Mountain View, CA, USA), as described previously [34, 35] or TeloPrime Full-Length cDNA Amplification Kit V2 (Lexogen, Austria) as directed. The full-length double-strand cDNA was amplified over 18 cycles. Then, the full-length double-strand RNA-Seq Libraries were amplified over 15 cycles and were prepared using the Nextera XT DNA Library Preparation Kit (Illumina, San Diego, CA, USA) for Illumina sequencing, after which library quality was further assessed using the TapeStation 4200 with High Sensitivity D1000 ScreenTape (Agilent Technologies, Santa Clara, CA). All libraries were quantified using the HS Qubit dsDNA assay (Thermo Fisher Scientific, Waltham, MA). All libraries were sequenced (2 × 75 bp) using Illumina NextSeq 500 (Illumina, San Diego, CA) according to the standard Illumina protocol. The FASTQ files were generated using the bcl2fastq software (Illumina). The FASTQ data is deposited in GEO (GSE189019).

### Data analysis

FASTQ files were checked using the FastQC software (version 0.11.7) [36] and aligned to the reference human genome (hg38) using HISAT2 (version 2.1.0) [37]. The StringTie algorithm (v.1.3.4d) [38] was then used with default parameter settings to assemble RNA-Seq alignments into annotated transcripts to estimate their expression using the UCSC annotated human genome (hg38) assembly file. Subsequently, the transcript expression was normalized using the transcripts per million (TPM) algorithm. For differential expression analysis, we used the R package (edgeR) [39]. For analysis and interpretation, we used SAMtools (v.1.9) [40], BEDTools (v.2.27.1) [41], Seqkit (version 0.13.2) [42], RSeQC (v.3.0.1) [43], and Ngsplot (v.2.6.3) [44]. For alternative splice events analysis, we used SplAdder software (v.2.4.2) with the UCSC annotated human genome (hg38) assembly file [23] and analysis approaches described previously [34, 45].

## Data Availability

The datasets (GSE189019) generated and/or analyzed during the current study are available in the Gene Expression Omnibus (GEO) database (https://www.ncbi.nlm.nih.gov/geo/).

## Abbreviations

PSI: Percent splicing in index
RNA-Seq: RNA sequencing
TSS: Transcription start site
